# Supplementary material for: Meta-Analysis and Gene Set Enrichment Relative to ER Status Reveal Elevated Activity of MYC and E2F in the “Basal” Breast Cancer Subgroup
Source: PLoS One. 2009 Mar 9;4(3):e4710. doi: 10.1371/journal.pone.0004710 (PMC2650420; doi:10.1371/journal.pone.0004710)
Supplement: Dataset S1 — GSEA results from first screen. The file contains a zipped archive of the HTML results of the second GSEA screen. Reports for enriched or depleted datasets may be accessed from the “index.html” link within the my_analysis.GseaPreranked.1219975950625.rpt.zip ZIP archive. Enrichment plots are available for each of the 50 most enriched and depleted gene sets. (13.32 MB ZIP) [file pone.0004710.s001.zip › my_analysis.GseaPreranked.1219975950625.rpt.zip!//YU_CMYC_UP.html]

Details for gene set YU\_CMYC\_UP[GSEA]

|  || Dataset | zSco.ERneg.to.ERpos\_collapsed |
| Phenotype | NoPhenotypeAvailable |
| Upregulated in class | na\_pos |
| GeneSet | YU\_CMYC\_UP |
| Enrichment Score (ES) | 0.7261426 |
| Normalized Enrichment Score (NES) | 2.3182633 |
| Nominal p-value | 0.0 |
| FDR q-value | 0.0 |
| FWER p-Value | 0.0 |
Table: GSEA Results Summary

  

Fig 1: Enrichment plot: YU\_CMYC\_UP      
 Profile of the Running ES Score & Positions of GeneSet Members on the Rank Ordered List

  

| PROBE | GENE SYMBOL | GENE\_TITLE | RANK IN GENE LIST | RANK METRIC SCORE | RUNNING ES | CORE ENRICHMENT || 1 | BUB1 |  |  | 41 | 6.445 | 0.0668 | Yes |
| 2 | GMNN |  |  | 206 | 5.302 | 0.1119 | Yes |
| 3 | BIRC5 |  |  | 229 | 5.191 | 0.1665 | Yes |
| 4 | CKS2 |  |  | 239 | 5.145 | 0.2215 | Yes |
| 5 | CLIC4 |  |  | 314 | 4.881 | 0.2689 | Yes |
| 6 | KIF20A |  |  | 318 | 4.874 | 0.3215 | Yes |
| 7 | CSDA |  |  | 337 | 4.835 | 0.3725 | Yes |
| 8 | ASPM |  |  | 359 | 4.767 | 0.4226 | Yes |
| 9 | CKS1B |  |  | 416 | 4.638 | 0.4686 | Yes |
| 10 | CCNB2 |  |  | 431 | 4.589 | 0.5173 | Yes |
| 11 | AURKA |  |  | 460 | 4.483 | 0.5637 | Yes |
| 12 | UCHL5 |  |  | 765 | 3.814 | 0.5822 | Yes |
| 13 | FDPS |  |  | 949 | 3.555 | 0.6070 | Yes |
| 14 | PLK1 |  |  | 988 | 3.499 | 0.6420 | Yes |
| 15 | E2F8 |  |  | 1122 | 3.334 | 0.6681 | Yes |
| 16 | BRCA2 |  |  | 1126 | 3.331 | 0.7040 | Yes |
| 17 | PCNA |  |  | 1346 | 3.087 | 0.7210 | Yes |
| 18 | CDKN3 |  |  | 1827 | 2.674 | 0.7139 | Yes |
| 19 | ECT2 |  |  | 2026 | 2.509 | 0.7261 | Yes |
| 20 | TSPAN4 |  |  | 3123 | 1.877 | 0.6640 | No |
| 21 | NUP54 |  |  | 3468 | 1.724 | 0.6568 | No |
| 22 | TOP2A |  |  | 3735 | 1.610 | 0.6543 | No |
| 23 | RACGAP1 |  |  | 4097 | 1.475 | 0.6431 | No |
| 24 | DTL |  |  | 4509 | 1.314 | 0.6264 | No |
| 25 | RRM1 |  |  | 4872 | 1.197 | 0.6122 | No |
| 26 | IDI1 |  |  | 6517 | 0.645 | 0.4955 | No |
| 27 | HMGN2 |  |  | 10916 | -1.492 | 0.1808 | No |
Table: GSEA details [plain text format]

  

Fig 2: YU\_CMYC\_UP: Random ES distribution      
 Gene set null distribution of ES for **YU\_CMYC\_UP**
